# New Genotype of *Yersinia pestis* Found in Live Rodents in Yunnan Province, China

**DOI:** 10.3389/fmicb.2021.628335

**Published:** 2021-04-15

**Authors:** Liyuan Shi, Jingliang Qin, Hongyuan Zheng, Ying Guo, Haipeng Zhang, Youhong Zhong, Chao Yang, Shanshan Dong, Fengyi Yang, Yarong Wu, Guangyu Zhao, Yajun Song, Ruifu Yang, Peng Wang, Yujun Cui

**Affiliations:** ^1^Yunnan Institute of Endemic Diseases Control and Prevention, Dali, China; ^2^State Key Laboratory of Pathogen and Biosecurity, Beijing Institute of Microbiology and Epidemiology, Beijing, China; ^3^School of Basic Medical Sciences, Anhui Medical University, Hefei, China

**Keywords:** *Yersinia pestis*, pathogenicity, low virulence, iron content, live rodent

## Abstract

Yunnan Province, China is thought to be the original source of biovar Orientalis of *Yersinia pestis*, the causative agent of the third plague pandemic that has spread globally since the end of the 19th century. Although encompassing a large area of natural plague foci, *Y. pestis* strains have rarely been found in live rodents during surveillance in Yunnan, and most isolates are from rodent corpses and their fleas. In 2017, 10 *Y. pestis* strains were isolated from seven live rodents and three fleas in Heqing County of Yunnan. These strains were supposed to have low virulence to local rodents *Eothenomys miletus* and *Apodemus chevrieri* because the rodents were healthy and no dead animals were found in surrounding areas, as had occurred in previous epizootic disease. We performed microscopic and biochemical examinations of the isolates, and compared their whole-genome sequences and transcriptome with those of 10 high virulence *Y. pestis* strains that were isolated from nine rodents and one parasitic flea in adjacent city (Lijiang). We analyzed the phenotypic, genomic, and transcriptomic characteristics of live rodent isolates. The isolates formed a previously undefined monophyletic branch of *Y. pestis* that was named 1.IN5. Six SNPs, two indels, and one copy number variation were detected between live rodent isolates and the high virulence neighbors. No obvious functional consequence of these variations was found according to the known annotation information. Among genes which expression differential in the live rodent isolates compared to their high virulent neighbors, we found five iron transfer related ones that were significant up-regulated (| log_2_ (FC) | > 1, p.adjust < 0.05), indicating these genes may be related to the low-virulence phenotype. The novel genotype of *Y. pestis* reported here provides further insights into the evolution and spread of plague as well as clues that may help to decipher the virulence mechanism of this notorious pathogen.

## Importance

The virulence mechanism of *Y. pestis* has always been the focus of scientists. Here by biochemical features, whole-genome sequences, and transcriptomes analysis, we found that the *Y. pestis* strains isolated from HQ live rodents belonged to a novel *Y. pestis* genotype. The iron transfer-related genes that were significantly up-regulated in the live rodent isolates, may be related to the low-virulence phenotype.

## Introduction

Plague is a fatal disease caused by *Yersinia pestis*, an enterobacteria that has caused three pandemics that have claimed millions of lives (Yang et al., 2011). Plague is an endemic disease that can form a natural focus in certain geographical and ecological environments, and, in particular, can be transmitted to humans living near natural foci through flea bites or airborne droplets (Schneider et al., 2014). In China, most of the known *Y. pestis* genotypes have high virulence. The exception is biovar Microtus, also known as 0.PE4 phylogroup (Cui et al., 2013) that is distributed mainly in Inner Mongolia and Qinghai Province, which has low virulence to humans and large mammals but high virulence to the voles *Lasiopodomys brandtii* and *Microtus fuscus* (Ji, 1988).

As the original source of the third plague pandemic (Morelli et al., 2010), Yunnan Province in China historically has a large area of natural plague foci. Three types of natural plague foci have been detected so far, namely the Yunnan domestic rodent plague foci, Yulong wild rodent plague focus (Lijiang City, hereafter referred to as “LJ”), and Jianchuan wild rodent plague focus (Jianchuan County) (Shi et al., 2018). The Yunnan domestic rodent plague foci are distributed mainly in the southwest, central, and eastern regions of Yunnan Province, whereas the two wild rodent plague foci are concentrated in northwestern Yunnan. Since the 1950s, active animal plague surveillance and prevention programs have been conducted annually in Yunnan (Song, 2009) and plague epidemics have been largely controlled, except from 1982 to 2007 when a plague epidemic reemerged and dozens of human plague outbreaks occurred across nearly half of Yunnan Province. Plague still remains endemic in some places (Shi et al., 2018). In Yunnan, *Y. pestis* strains have usually been isolated from rodent corpses and their fleas during plague outbreaks, and occasionally from live rodents (Chen, 2019). Only rarely have *Y. pestis* strains been isolated from healthy live rodents, and no dead (rodents that were found dead) or diseased animal was found around the investigation area.

Heqing County (hereafter referred to as “HQ”), which is adjacent to the LJ and Jianchuan wild rodent plague foci, is in the northern Dali Bai Autonomous Prefecture, Yunnan, China. Historical documents indicate there were three major epidemics in HQ that were suspected to be plague. They occurred in 1772–1773, 1776–1796, and 1879–1888, and about 15,000 people died (Guo et al., 2018). In 1985, the plague F1-antibody was detected in four *Y. pestis*-positive serum samples from dogs and rodents in HQ; however, no confirmed epidemic was reported until 2017. On 17 April 2017, two *Y. pestis* strains were isolated from two of 87 live rodents in Damachang Village, HQ during routine plague surveillance, but no dead or diseased rodents were found (the positive rate was 2.3%) (Guo et al., 2018). Accordingly, we inferred that a rodent plague epidemic had occurred in the HQ region. To determine the intensity and scope of the rodent plague in HQ, we performed epidemiological investigations in Damachang Village and its surrounding areas between 24 April and 28 May 2017. We isolated eight more *Y. pestis* strains from five live rodents and three fleas, and different with previous plague outbreaks, still no dead animal was found in the surrounding areas. Therefore, according to the results of epidemiological investigation, we inferred that the *Y. pestis* strains in HQ may have relatively lower virulence to local rodent compared with the other isolates in natural plague foci of Yunnan. In this study, we reviewed the epidemiological data in HQ, analyzed the histopathology of the infected rodents, and determined the biochemical phenotypes, genomes, and transcriptomes of the *Y. pestis* strains, to gain insights into the properties of the newly isolated *Y. pestis* strains.

## Materials and Methods

### *Y. pestis* Isolate Collection

Two HQ *Y. pestis* strains were collected in April, 2017 during daily surveillance, and eight more ones were isolated from rodents and their body fleas that were captured in the following large-scale field investigation (Guo et al., 2018). The mouse traps were set near the burrows of host animal, and in every day the traps were checked for 3 days, as well as to search for dead or diseased animals around the investigation area. After fumigating by diethyl ether, the body hairs of the captured animals were combed and body fleas were uncovered. Fleas were collected in normal saline, and their gastric contents were inoculated on plates. Blood samples from dogs and rodents were centrifuged to separate the serum for F1-Antibody detection by indirect hemagglutination test (IHA). Samples of liver and spleen from the captured live rodents, and samples of gland, heart, lung, liver, spleen, bone marrow from dead rodents were used to be cultured first and then be detected the F1 antigen by reverse indirect hemagglutination test (RIHA).

Ten *Y. pestis* isolates were collected from nine rodents and one parasitic flea in four villages in LJ during 2006–2018. Among the nine strains detected, seven strains were isolated from rodent corpses, and two strains were isolated from live rodents during the plague outbreak (Table 1).

**TABLE 1:** Background information of the 20 *Y. pestis* isolates in Yunnan.

**Isolates**	**City**	**County**	**Address**	**Date**	**Host**	**Isolation source**	**Host state**
LJ14	Lijiang City	Yulong County	Luzi Village	2006/11/14	*N. specialis specialis*	proventriculus and midgut	——
LJ1367	Lijiang City	Yulong County	Luzi Village	2006/11/26	*A. chevrieri*	liver and spleen	dead
LJ485	Lijiang City	Yulong County	Luzi Village	2006/11/14	*Rattus nitidus*	liver and spleen	dead
LJ935	Lijiang City	Yulong County	Danqian Village	2014/11/16	*Eothenomys spp.*	liver and spleen	live
LJ00	Lijiang City	Yulong County	Luzi Village	2017/4/14	*Eothenomys spp.*	liver and spleen	dead
LJMS	Lijiang City	Gucheng District	Mushu Village	2017/4/11	*A. chevrieri*	liver and spleen	dead
LJ179	Lijiang City	Yulong County	Jizi Village, Taian Township	2018/4/13	Rat (Unknown)	liver and spleen	dead
LJ236	Lijiang City	Yulong County	Jizi Village, Taian Township	2018/4/17	*Eothenomys spp.*	liver and spleen	live
LJ258	Lijiang City	Yulong County	Jizi Village, Taian Township	2018/4/21	*A. chevrieri*	liver and spleen	dead
LJ261	Lijiang City	Yulong County	Jizi Village, Taian Township	2018/4/24	*A. chevrieri*	liver and spleen	dead
HQ16	Dali Prefecture	Heqing County	in the north of Damachang Village	2017/4/17	*E. miletus*	liver and spleen	live
HQ21	Dali Prefecture	Heqing County	in the north of Damachang Village	2017/4/17	*E. miletus*	liver and spleen	live
HQ32	Dali Prefecture	Heqing County	in the north of Damachang Village	2017/4/23	*A. chevrieri*	liver and spleen	live
HQ112	Dali Prefecture	Heqing County	in the north of Damachang Village	2017/4/23	*E. miletus*	liver and spleen	live
HQ125	Dali Prefecture	Heqing County	in the west of Damachang Village	2017/4/30	*N. specialis specialis*	proventriculus and midgut	——
HQ139	Dali Prefecture	Heqing County	in the west of Damachang Village	2017/4/30	*Ctenophthalmus quadratus*	proventriculus and midgut	——
HQ146	Dali Prefecture	Heqing County	in the west of Damachang Village	2017/4/28	*E. miletus*	liver and spleen	live
HQ153	Dali Prefecture	Heqing County	in the west of Damachang Village	2017/4/28	*N. specialis specialis*	proventriculus and midgut	——
HQ161	Dali Prefecture	Heqing County	in the west of Damachang Village	2017/4/28	*E. miletus*	liver and spleen	live
HQ164	Dali Prefecture	Heqing County	in the west of Damachang Village	2017/4/28	*E. miletus*	liver and spleen	live

*A total of 8 years of rodent epidemics occurred in Lijiang from 2006 to 2018. Except for the strain isolated in 2006, 2017, and 2018, only nucleic acid positive was found in other years. The strain isolated in 2014 was accidentally isolated from the intestines of a captured live rodent.*

### DNA Extraction

We selected 10 HQ isolates (*Y. pestis* strains were isolated from seven live rodents and three fleas) and 10 high virulence *Y. pestis* strains that were isolated from LJ (the adjacent city) for DNA extraction. The extracted DNA was prepared for sequencing after recovery (the preserved *Y. pestis* strains were inoculated in added blood (rabbit) LB medium and cultured at 28°C for 24 h), activation, and proliferation (the activated strains were inoculated in the new medium and the culture conditions were same as before). A QIAGEN DNeasy Blood & Tissue (No. 69506) kit (QIAGEN Shanghai, China) and a Promega Wizard Genomic DNA Purification Kit (A1120) were used to extract the DNA for next-generation sequencing and third-generation sequencing, respectively.

### Whole-Genome Sequencing and Assembly

Whole-genome sequencing was performed on an Illumina MiSeq platform with 150-bp paired-end libraries, and the raw sequencing reads were trimmed using Trimmomatic (Bolger et al., 2014). After filtering, clean reads with target mean coverage >80 × and quality score >20 remained. The clean reads were assembled *de novo* using SPAdes (Bankevich et al., 2012). Isolates LJ935 and HQ16 were also sequenced on a Pacific Biosciences (PacBio) RS II platform at the Beijing Genomics Institute (BGI, Shenzhen, China) to obtain the complete genome sequence. About 100,000 PacBio long reads were generated, which corresponds to 158 × coverage of the estimated *Y. pestis* genome. The average read length was 6,774 and 9,142 for LJ935 and HQ16, respectively. Sequence reads were assembled using Unicycler^1^.

### Phylogeny Reconstruction

We sequenced the genomes of 10 HQ isolates and 10 LJ isolates (Yulong wild rodent plague focus), and compared them with 368 previously published genomes that represented the global diversity of *Y. pestis* (Table 1 and Supplementary Table 1). All assemblies were aligned against a reference genome of strain CO92 (accession no. NC_003143.1) using MUMmer (v3.0) (Kurtz et al., 2004) to generate whole-genome alignments and to identify single nucleotide polymorphisms (SNPs) in the core genome. The raw sequencing reads were mapped to the assemblies to evaluate the SNP accuracy using SOAPaligner (Li R. et al., 2009) as described previously (Yang et al., 2019). This process excludes unreliable SNPs located in repeated regions with low-quality scores (<20) or supported by few reads (<10 paired-end reads). Maximum likelihood phylogeny was inferred using PhyML (Guindon et al., 2010) from an alignment of core genome SNPs using the genome of strain CO92, which was the phylogenetically most related genome to our isolates at the time of analysis. The phylogeny was rooted using phylogroup 0.PE7 genomes as outgroups. Confidence in individual branching relationships was assessed using 100 bootstrap replicates and the tree was visualized using FigTree software^2^. Because the core genomes in different datasets were variable, which could affect SNP calling, we recalled SNPs for the newly sequenced genomes using the same pipeline to obtain higher resolution. We identified 52 high quality SNPs from the datasets of the 20 new isolates. These SNPs were used as the character set in the Applied Maths Bionumerics 6.6 software^3^, which was used to construct a minimum spanning tree (MSTree) with hypothetical intermediate nodes. The MSTree was fully parsimonious for all 20 *Y. pestis* isolates and was confirmed using *Y. pestis* Z176003 (1.IN2 phylogroup strain, accession no. NC_014029.1) as the outgroup.

### Typing by Multilocus Variable-Number Tandem-Repeat Analysis

The 25 variable number of tandem repeat (VNTR) loci initially reported by Pourcel et al. (2004) were used to type the 20 newly isolates. Based on the VNTR locus information, we extracted the VNTR profiles of the HQ and LJ isolates and compared them with the VNTR profiles of three Xinjiang strains of 1.IN4 (XJ2651, XJ2650, XJ2656) reported previously (Zhang et al., 2018).

### Genomic Variation Detection

We aligned the 20 newly isolates to the selected reference genome, LJ935, using BWA software (v0.7.17) (Li and Durbin, 2010) to identify indels <50-bp long, and visualized them using SAMtools (Li H. et al., 2009). The indel loci and the 300-bp sequences flanking the indels were extracted and compared with the nucleotide sequences in the NCBI database^4^ to obtain the alleles. The annotation information was obtained according to the position of the alleles (Cui et al., 2020). All the assemblies were used in Prokka (Seemann, 2014) for gene annotation, and the annotation results (gff3 files) were used in Roary (Page et al., 2015) to identify the pan-genome and generate a matrix of the presence/absence of the genes in each sample as described previously (Yang et al., 2019). Gene rearrangements in the two complete maps (LJ935 and HQ16) and assemblies were identified by performing a multiple genome alignment with rearrangements using Mauve^5^ (Darling et al., 2004). Finally, a copy number variation (CNV) analysis was performed using the pipeline described in see section “Genomic Variation Detection.” Briefly, the paired-end reads were mapped to the LJ935 genome using BWA (Li and Durbin, 2009). The CNVs were predicted and corrected by CNGpro (v1.25.0) in each 100-bp window (Brynildsrud et al., 2015; Brynildsrud, 2018). In the data statistical analysis, all the results were classified by region (Lijiang or Heqing) and host state (dead or live rodent).

### RNA Extraction and Sequencing

Total RNA was isolated from the four strains isolated from HQ (HQ16 and HQ32) and LJ (LJ236 and LJMS) using the Qiagen RNeasy^®^ Mini kit (No. 74104) and RNAprotect Bacteria Reagent (No. 76506), and purification was performed according to the manufacturer’s instructions. Sequencing libraries were generated using NEBNext^®^ UltraTM RNA Library Prep Kit for Illumina^®^ (NEB, United States) following manufacturer’s recommendations and index codes were added to attribute sequences to each sample. The prepared libraries were sequenced on an Illumina Novaseq platform and 150-bp paired-end reads were obtained. Read quality statistics were evaluated using FastQC^6^.

### RNA-Seq Data Analysis

Paired-end raw reads were trimmed using Trimmomatic (Bolger et al., 2014). Aligned reads were annotated and counted using HTSeq (v0.6.1) (Anders et al., 2015). Differentially expressed genes (DEGs) between the two groups were detected using DESeq2 with the following cutoffs: Benjamini-Hochberg adjusted *P*-value (p.adjust) < 0.05 and absolute log_2_(fold change) >1 (Deng et al., 2019). The Pearson correlation coefficient (*R*) of RNA-seq replicates was computed based on the DESeq2 regularized logarithm (rlog) normalized read counts (Love et al., 2014). The DEGs were annotated based on reference genome of *Yersinia pestis* YN1683 (accession no. NZ_ADTD00000000.1). Rockhopper (McClure et al., 2013) was used to identify new intergenic region transcripts, and Blastx was compared with the Non-Redundant Protein Sequence Database to annotate the newly predicted transgenic regions. The DEGs were functionally annotated by sequence homology searches against the Kyoto Encyclopedia of Genes and Genomes (KEGG) pathway database (Kanehisa et al., 2010) and the Swiss-Prot database^7^. In this study, we focused on quantifying only known mRNA sequences, so unknown genes or isoforms were not considered (Kim et al., 2020).

### Pathological-Anatomic Observation, and Liver and Spleen Tissue Sections

The health status of the captured rodents in HQ was judged by observing their manner, reaction, activity, and hair as described previously (Guo et al., 2018). During the dissection, the rodent lymph nodes, heart, lung, liver, and spleen tissues were examined pathologically, and subcutaneous hemorrhage, and pleural and peritoneal effusion were noted. The spleen and liver samples were sectioned and stained with hematoxylin and eosin and observed under a microscope.

### Biochemical Phenotypic Determination

Three isolates of HQ were selected randomly and compared with their phylogenetically nearest neighbors (three isolates from LJ), one strain from each of three other distinct *Y. pestis* lineages (Yunnan domestic rodent strain, Jianchuan wild rodent strain, and EV76 strain), and one *Yersinia pseudotuberculosis* strain. The following biochemical tests were performed: sugar–alcohol assays (maltose, glycerol, melibiose, arabinose, and rhamnose); nitrate reduction test; V virulence factor test, pigmentation (*pgm*) virulence factor test, and nutritional requirement detection. For the sugar–alcohol assays, *Y. pestis* strains were diluted and dripped into fermentation tubes, which were cultured at 37°C for 7 days. For the nitrate reduction test, the *Y. pestis* strains were cultured in nutrient liquid containing nitrate at 28°C for 48 h, and the biochemical reactions were observed after adding two Gliss reagents. For the V virulence factor test, diluted bacterial suspensions (10^4^ CFU per ml) were cultured in calcium-deficient medium or LB medium at 37°C for 72 h, then transferred to 28°C for 24 h. The determination of V was according to the number of bacterial colonies on the two mediums. For the *pgm* virulence factor test, the bacterial suspension was diluted to the desired bacterial titer (10^3^ CFU per ml) and evenly coated on Congo red plates, then incubated at 28°C for 3 days. For the nutritional requirement detection, the bacteria were incubated in glutamate-deficient medium or phenylalanine-deficient medium and cultured at 28°C for 4 days, then colony growth was observed.

## Results

### Epidemiological Investigation and Bacteria Isolation in Heqing County, and Pathological-Anatomic Observation in Heqing Live Rodents

**FIGURE 1:**
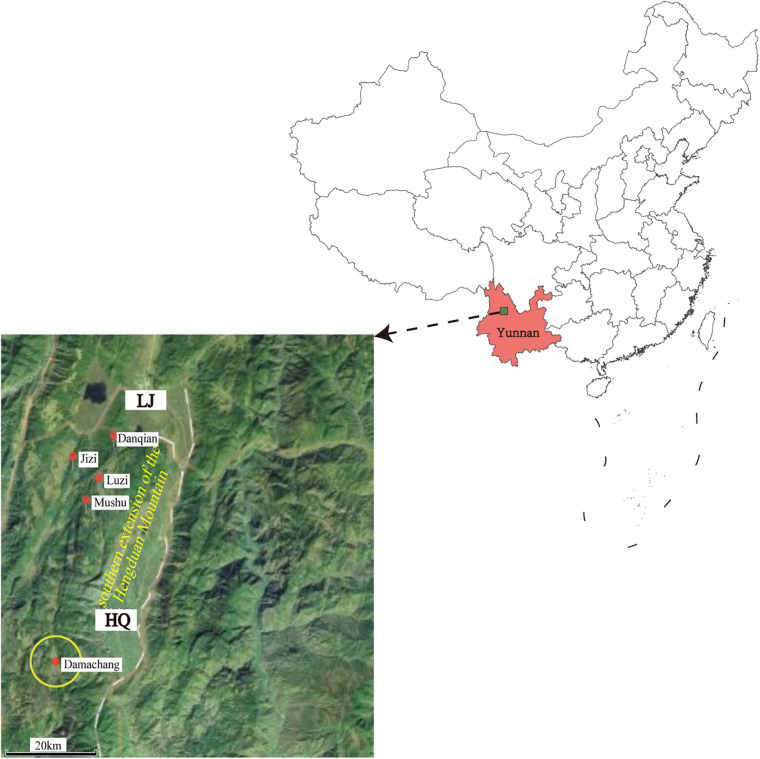
Geographic locations of the new isolates in Heqing County and neighboring Lijiang City. Red circles indicate the locations of the newly sequenced isolates described in this study. The size of the yellow circle indicates the scope of the epidemiological investigation. The map was created using ggmap (Kahle and Wickham, 2013) based on the public geographical data downloaded from OpenStreetMap (http://openstreetmap.org).

The field investigation of the HQ plague epidemic was conducted from 17 April to 28 May 2017, which surrounded Damachang Village, HQ County within a radius of 5 km. Six villages were included and 7,090 individuals were checked through household inspection and quarantine, and no one with high fever or who was seriously ill was found (Guo et al., 2018). In total, 354 live rodents were captured and 242 body fleas were collected for *Y. pestis* detection, and no dead or diseased animals were found around the investigation area. Ten *Y. pestis* strains were isolated in HQ; six isolates were from *Eothenomys miletus* (*E. miletus*), one was from *Apodemus chevrieri* (*A. chevrieri*), one was from *Ctenophthalmus quadratus*, and two were from *Neopsylla specialis specialis* (*N. specialis specialis*). The surveillance found that the rodent plague had appeared only in the cultivated land, with a radius of 300 meters around the earliest isolation site (Figure 1). The surveillance of plague in Yunnan was performed according to standard protocol in National Scheme of Plague Surveillance of China. More details about the plague surveillance programmes are provided in http://www.gov.cn/yjgl/2005-08/30/content_28245.htm.

**FIGURE 2:**
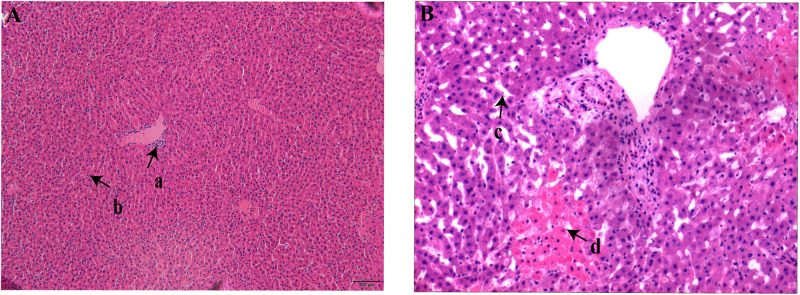
Liver section from Heqing County live rodents positive for bacterial culture. **(A)**, Lymphocyte infiltration in the portal area (a) and hepatocyte cords (b). **(B)** Hepatic sinusoids (c) were significantly dilated and congested and there was patchy necrosis of hepatocytes (d).

All the rodents captured in HQ were live rodents and they remained healthy with no obvious clinical signs of disease; that is, they exhibited normal daily activity, had shiny hair, bright eyes, and agile movements, which indicated a healthy state. Anatomical observation showed that the rodents had slight subcutaneous hyperemia, normal sized glands, and a small amount of red pleural and peritoneal effusion. The colors of the heart, lung, liver, and spleen tissues were normal, and there was no swelling, nodules, or necrosis in these organs. Three rodents positive for bacterial culture were selected randomly and their livers and spleens were sectioned. Microscopic examination of these sections revealed lesions in different levels of the liver tissue. Some lesions were lymphocyte infiltrations in portal areas and hepatocyte cords, and some were both lymphocyte infiltration and patchy necrosis of hepatocytes, and dilation and congestion of hepatic sinusoids (Figure 2). No obvious pathological changes were found in spleen sections. Pathological–anatomic observation of the liver and spleen tissue sections suggested that the *Y. pestis* isolated from HQ might have low virulence for *E. miletus* and *A. chevrieri*.

### A Novel Phylogenetic Branch of *Y. pestis*

**FIGURE 3:**
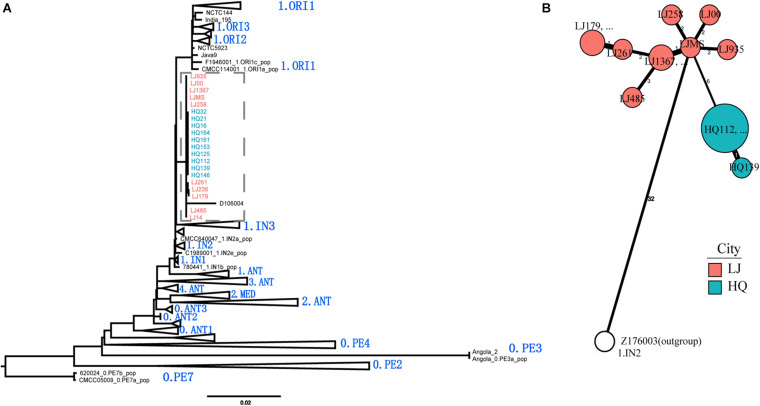
Phylogenetic tree of 388 *Y. pestis* genomes and phylogeny of the new 1.IN5 phylogroup. **(A)** Maximum likelihood phylogenetic tree for *Y. pestis* consisting of 368 public genomes and 20 newly sequenced isolates. The tree was built using PhyML. A total of 5,458 polymorphic sites were used to construct the tree, which was visualized and edited using FigTree. Main branches/sub-branches were collapsed for clarity. **(B)** Minimum spanning phylogeny of 20 *Y. pestis* strains in Heqing County (HQ) and Lijiang City (LJ), based on 52 SNPs. The labels are the strain IDs. Red circles indicate the LJ isolates; blue circles indicate the HQ isolates; white circle indicates the outgroup strain.

To define the phylogenetic position of HQ live rodent isolates in *Y. pestis* genealogy, we sequenced the genomes of 10 HQ isolates and 10 LJ isolates (Yulong wild rodent plague focus), and compared the sequences with 368 published genome sequences that represented the global diversity of *Y. pestis* (Table 1 and Supplementary Table 1). We built a maximum likelihood tree of 388 *Y. pestis* genomes on the basis of 5,458 SNPs. The HQ and LJ genomes grouped together in phylogenetic tree and formed a monophyletic lineage that was located between the known phylogroups, 1.ORI and 1.IN3 (Figure 3A). In a previously reported phylogenetic tree that was based on the diversity of 25 VNTR loci, three *Y. pestis* strains that were isolated from Xinjiang Autonomous Region, China also were located between 1.ORI and 1.IN3 and were attributed to 1.IN4 group (Zhang et al., 2018). However, by comparing the VNTR profiles of the HQ and LJ isolates with the VNTR profile of the 1.IN4 strains, we found at least 14 different VNTR loci among them (Supplementary Table 2). Therefore, the genetic evidence indicated that the HQ and LJ strains formed a new phylogenetic group, which was designated as 1.IN5. Notably, the 10 HQ isolates (all from live rodents) were clustered together (Figure 3B), composing a unique genotype within the 1.IN5 group. Although all 10 LJ genomes were in the 1.IN5 group, they were isolated mainly from rodent corpses, suggesting the LJ strains had higher virulence than the HQ strains.

### Comparison of Biochemical, Genomic, and Transcriptomic Characteristics Between Live Rodent Isolates and Isolates With Higher Virulence

#### Biochemical Characteristics

To characterize the phenotypes of the HQ isolates, we determined various biochemical characteristics. The results showed that maltose, glycerol, and arabinose fermentation, nitrate reduction, and *pgm* and V virulence factors were positive, whereas rhamnose and melibiose fermentation were negative, and the strains were unable to grow on phenylalanine-deficient medium (Table 2). We also determined and compared the phenotypes of the LJ strain, the phylogenetically nearest neighbor of the HQ isolates. We selected one strain from each of other three distinct *Y. pestis* lineages (Jianchuan wild rodent strain, Yunnan domestic rodent strain, and EV76 strain), and one strain from *Yersinia pseudotuberculosis*. The biochemical characteristics of the HQ isolates were consistent with those of the LJ wild rodent isolate. However, the biochemical characteristics from other group of strains exhibited differences from that of the HQ isolates. That is, Jianchuan wild rodent strain was maltose^–^, Yunnan domestic rodent strain was glycerol^–^, EV76 strain was glycerol^–^ and it lacked the *pgm* virulence factor, and *Yersinia pseudotuberculosis* strain was rhamnose^+^, melibiose^+^, *pgm*^–^, V^–^, and it grew normally on phenylalanine-deficient medium.

**TABLE 2:** Biochemical characteristics in live rodent isolates.

**Strain**	**Nutrients fermentation**					**Nitrate**	***pgm***	**V**	**Glutamate-deficient medium**	**Phenylalanine-deficient medium**
	**Maltose**	**Rhamnose**	**Arabinose**	**Melibiose**	**Glycerol**					
HQ32/HQ112/HQ153 (Heqing)	+	−	+	−	+	+	+	+	+	−
LJ485/LJ00/LJMS (Lijiang)	+	−	+	−	+	+	+	+	+	−
JC1332 (Jianchuan)	−	−	+	−	+	+	+	+	+	−
BN2636 (Yunnan domestic rodent strain)	+	−	+	−	−	+	+	+	+	−
EV76	+	−	+	−	−	+	−	+	+	−
PST-1 (*Yersinia pseudotuberculosis*)	+	+	+	+	+	+	−	−	+	+

*“+” and “−” in the rightmost two columns were determined based on growth of colonies and no growth of colonies on media, respectively.*

#### Genomic Variations

**FIGURE 4:**
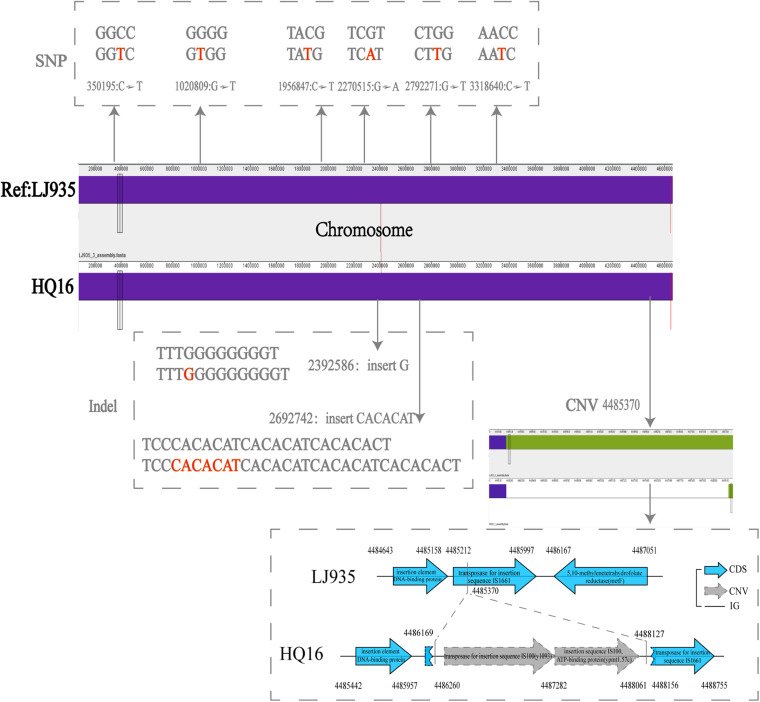
Distribution of SNPs, indels, and CNVs in the genomes of Heqing County (HQ) and Lijiang City (LJ) strains. The LJ935 genome was used as the reference. Dotted frames indicate different variations; red letters indicate the base sequences of the variation; gray numbers indicate the variation sites.

To detect the unique genetic variations in the HQ strains, we compared their genomes with the genomes of high virulence LJ strains, and explored five types of genome variations, namely SNPs, indels, gene presence/absence, gene rearrangement, and CNVs. Six high quality SNPs, two indels, and one CNV were identified between the two groups of genomes. No gene gain/loss or genome rearrangement events were identified by comparing both the complete maps and assemblies (Figure 4 and Supplementary Table 3). Among the six SNPs, three were intergenic, two were non-synonymous SNPs that were located in genes encoding a putative membrane protein and alpha-galactosidase, and one was a synonymous SNP in a gene encoding the high-affinity branched-chain amino acid transport ATP-binding protein. The two indels were insertions in intergenic regions and were not associated with annotated products. Compared with the LJ genomes, the genomes of the HQ live rodent isolates had one more copy of IS100 (Table 3), an insertion sequence element with multiple copies in the *Y. pestis* genome. Interestingly, the additional IS100 interrupted IS1661, another type of insertion sequence element, in all the HQ strains (Figure 4 and Supplementary Figure 1). It was difficult to find the functional consequences of these genetic variations using currently available genome annotations.

**TABLE 3:** An annotation on 1959 bp IS sequences.

Length*	Type*	**Gene**	**Locus**	**Product**
91	IG	–	–	–
1023	CDS	*y1093*	YPO0924	transposase for insertion sequence IS100
780	CDS	*ypmt1.57c*	YPO0923	insertion sequence IS100, ATP-binding protein
66	IG	–	–	–

* HQ16 was used as reference.

#### Transcriptome Analysis

**FIGURE 5:**
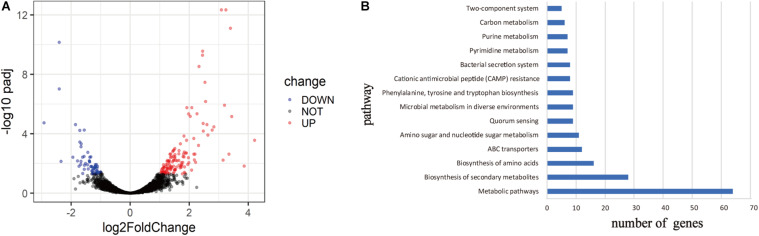
Differentially expressed genes (DEGs) between live rodent isolates (HQ) and the high virulence isolates from wild rodents (LJ). **(A)** Volcano plots of the DEGs between the HQ and LJ isolates. Red and blue spots indicate the DEGs; black spots indicate genes that were not differentially expressed. **(B)** Kyoto Encyclopedia of Genes and Genomes (KEGG) functional annotation of the DEGs. The top 14 enriched pathways are shown; pathways with less than five genes are not shown.

To explore the mechanism of virulence differences between live rodent isolates and the high virulence isolates from wild rodents, we selected two strains from HQ (HQ16 and HQ32) and two strains from LJ (LJ236 and LJMS), for gene expression analyses by RNA-seq. A total of 191 DEGs were found (absolute log_2_ (fold change) >1; p.adjust < 0.05); 133 from the HQ strains were up-regulated and 58 were down-regulated compared with their expression in the LJ strains (Supplementary Table 4, Supplementary Figure 2, and Figure 5A). To predict the biological function of the DEGs, we mapped them into the KEGG database to identify genes involved in pathways (Kim et al., 2020) and annotated the identified genes by aligning them to the Swiss-Prot database. The DEGs were assigned to 14 KEGG pathways (Figure 5B), including Metabolic pathways, Biosynthesis of secondary metabolites, Biosynthesis of amino acids, and ABC transporters; among them, Metabolic pathways was the most enriched, followed by Biosynthesis of secondary metabolites. The transcripts of nine genes that carried variations were not detected in the RNA-seq data. For the four genes that were detected in the RNA-seq data, the expression differences between the two groups were not significant.

Notably, we found six DEGs that were iron transport related; five were up-regulated and one was down-regulated in the HQ isolates compared with the LJ isolates (Table 4). Then, we checked all 3,298 genes in the RNA-seq data (genes with no reads coverage were excluded) and found 54 that were iron-related, which had no statistical difference compared with six iron-related genes in 191 DEGs (*X^2^* = 1.608, *P* > 0.05). However, we cannot rule out that the transcription of iron-related genes may be related to the different phenotypic pattern between the HQ and LJ *Y. pestis* isolates.

**TABLE 4:** Expression of six iron transport-related genes in live rodent isolates (HQ) and the high virulence isolates from wild rodents (LJ).

**Gene ID**	**Log_2_FC**	***P***	**P. adj**	**Length**	**FPKM**				**IfcSE**	**Stat**	**Product**
					**HQ16**	**HQ32**	**LJ236**	**LJMS**			
DW34_RS14460	1.441107	1.94E-05	0.001561	1066	461.3234	457.0018	161.0337	137.2176	0.337369	4.271608	Fe-S cluster assembly protein SufD
DW34_RS14450	1.51023	6.36E-05	0.003555	1512	489.5513	292.1406	142.4419	98.94075	0.37765	3.999023	Fe-S cluster assembly protein SufB
DW34_RS14455	1.300887	0.000168	0.006997	747	532.9319	583.8491	219.1628	180.9808	0.345665	3.763427	Fe-S cluster assembly ATPase SufC
DW34_RS19920	1.251685	0.000925	0.022427	954	468.5238	292.5187	154.1146	126.611	0.377872	3.312455	siderophore ABC transporter substrate-binding protein
DW34_RS18340	1.086678	0.002488	0.045835	1845	165.0024	189.7152	68.05829	79.2813	0.359252	3.024831	IucA/IucC family siderophore biosynthesis protein
DW34_RS10775	−1.15119	0.002865	0.049475	1385	186.7237	372.4675	557.7464	543.2669	0.386067	−2.98183	TonB-dependent siderophore receptor

*Log_2_FC, Heqing (HQ) vs. Lijiang (LJ) log_2_ fold change value; P. adj, Benjamini-Hochberg adjusted *P*-value; FPKM, fragments per kilobase of exon model per million mapped fragments; lfcSE, Log_2_FC standard error; Stat, Wald statistic value.*

## Discussion

In this study, we performed an epidemiological investigation of the newly defined HQ wild rodent plague focus, and determined the phenotypic, genomic, and transcriptomic characteristics of HQ *Y. pestis* strains. On the basis of our previously epidemiological investigation and bacterial culture results (Guo et al., 2018), we proposed that *E. miletus* and *A. chevrieri* were the dominant rodent hosts, and *N. specialis specialis* was the main vector in the new natural plague focus (*Ctenophthalmus quadratus* had no media effectiveness) (Liang et al., 1996). The *Y. pestis* isolates from HQ belong to the 1.IN5 phylogroup that also contained LJ wild rodent isolates on the deeper branches in the phylogenetic tree (Figure 3B), which suggested that the HQ isolates probably originated in the LJ region. Both HQ and LJ are located in the southern extension of the Hengduan Mountains, with a geographical distance of 35 km them (Figure 1). Therefore, the emergence of the HQ wild rodent plague focus may be a consequence of the southward expansion of the LJ plague focus.

Although geographically adjacent, the epizootic pattern of plague in HQ was different from that in LJ or other natural plague foci in Yunnan. Usually, a plague epizootic is noticed in routine surveillance when an unexplained rodent corpse is found and *Y. pestis* strains are successfully isolated. Occasionally *Y. pestis* strains have been isolated from live diseased rodents during the large-scale investigation. However, in the HQ plague focus, all the *Y. pestis* strains were detected in live rodents or their parasitic fleas even in the expanded investigation. Anatomical observations showed that the HQ live rodents had slight subcutaneous hyperemia and a small amount of red pleural and peritoneal effusion, and no organ lesions visible to the naked eyes were found. Under a microscope the rodent liver samples had different levels of pathological changes, but no obvious pathological changes were found in the spleen samples. There are several explanations for why *Y. pestis* strains were found in physically well live rodents. First, the sensitivity to *Y. pestis* varied widely among host individuals in HQ, as was found for great gerbils in the Xinjiang Junggar Basin focus (Zhang et al., 2012). However, according to our knowledge, the odds are rare that during an epizootic in known natural plague foci, all *Y. pestis* strains would be isolated from rodents that had no obvious clinal symptoms, and not a single dead rodent would be found. Second, the rodents may not have had sufficient time from capture-to-dissection to develop clinical symptoms or die. However, this scenario also is unlikely because the large-scale investigation in HQ lasted for more than 1 month, and no dead or diseased animals were found in surrounding areas. Therefore, we supposed that the virulence of *Y. pestis* isolated from the HQ live rodents was attenuated in their local hosts.

Many factors can affect bacterial virulence, such as host state (Atilano et al., 2014), local climate (Friman et al., 2011), or microecology (bacteriophage). Temperate bacteriophages enhance or weaken the virulence of bacteria through lysogenic conversion and transduction (Argov et al., 2017; Fortier, 2017), and lytic bacteriophages allow strains with reduced virulence to reproduce by exerting selection pressure on bacteria, which may attenuate the virulence of bacteria (Leon and Bastias, 2015). To infer the mechanism for the virulence difference between the HQ and LJ isolates, we compared the genomic variations and transcriptomes between the two groups. Although 13 variations were identified, it was difficult to associate these variations with the virulence phenotypes based on known annotations (Supplementary Table 3 and Table 3). Notably, six of the detected DEGs were iron transport related and five were significantly up-regulated in HQ compared with LJ. Iron plays an important role in bacterial growth by promoting various biosynthetic processes, such as electron transport, oxidation-reduction, and nucleotide biosynthesis (Cai et al., 2016). Sufficient iron not only supports the complete surface structure of bacteria to maintain high invasiveness, but also affects the expression of iron-regulated virulence factors to maintain bacteria with high pathogenicity (Lv et al., 2012). Efficient iron acquisition systems are critical to the ability of *Y. pestis* to infect, spread, and grow (Schaible and Kaufmann, 2004). Therefore, if the iron uptake mechanism is disturbed during the bacterial growth or infection process, the pathogenicity of the bacteria to humans or other animals may be affected. Here, we propose two hypotheses for the significant changes of the iron-related genes expression in the HQ strains. First, it might be related to the compensatory mechanism of *Y. pestis* (Burgos and Schmitt, 2012). For example, in iron-deficient conditions, when the ABC transporter lipoproteins Pia or Piu may lose their iron transfer function, but the loss can be compensated by another transporter; however, when both transporters show loss-of-function at the same time, bacterial survival and virulence is seriously affected (Romero-Espejel et al., 2013). The HQ isolates may have a similar compensation mechanism. The absorption of low concentration iron by bacteria is through an active transport system in which TonB-dependent transporters (TBDTs) efficiently bind to iron-containing substrates in a form that does not require energy. After binding to the substrate, the TBDT conformation changes; then, TBDT interacts with TonB through the conserved region of the “TonB box,” which produces enough energy to complete the transport (Postle, 2007). More than 25% of Gram-negative bacteria were found to have two or more TonB systems (Chu et al., 2007). Multiple TonB systems that transport the same substance may be related to their living environment. When adapting to the environment, one of the TonB systems may change in response to the conditions. Under adverse environmental conditions, TonB systems could work together or, if one type of TonB loses its function, another TonB could compensate (Liao et al., 2015), resulting in compensatory transport and changes in *tonB* gene expression. The iron uptake process in *Y. pestis* also involves TBDTs for translocation across the outer membranes (Nairz et al., 2010; Schalk and Guillon, 2013). Second, the significant up-regulation of iron-related genes in the HQ strains might be related to characteristics of the rodent host (Suchdev et al., 2017) or to environmental factors in HQ. LJ and HQ are located in the southern extension of the Hengduan Mountains; the average elevation of LJ is 2,700 meters, whereas the HQ is closer to the southern end of the mountain range with an average elevation of 2,900 meters. These altitude differences result in different niches for *Y. pestis*. We suspect that the iron content or oxygen concentration in the HQ host or environment might be lower than those in LJ. To survive and reproduce (Holden et al., 2009) in HQ, *Y. pestis* needed to improve its ability to acquire iron from the outside, resulting in the higher expression of iron-related genes in HQ compared with LJ.

## Conclusion

The discovery of a new lineage of 1.IN5 enriches our knowledge of the global diversity of *Y. pestis*, and provides a foundation for further exploration of the evolution before the shaping of 1.ORI phylogroup that led to the third plague pandemic. Our results indicate that the Yulong wild rodent plague focus (LJ) is expanding southward along the Hengduan mountain range, therefore enhanced surveillance should be performed in HQ and surrounding regions. The unusual pattern that no sick/dead animals were found during the HQ plague epizootic suggested that *Y. pestis* strains were attenuated to their host in the local region. Unfortunately, because of the disposal procedure of plague epidemic, the captured live rodents could not be raised and observed after capture, and because of the biosafety requirements, the animal virulence test could not be performed in a local Yunnan laboratory. Although the up-regulation of the iron-related genes in the HQ strains provide clues for the unusual pattern, quantitative experiments on animal virulence, molecular mechanism studies, and comprehensive ecological investigations are needed to further understand the reduced virulence of the *Y. pestis* strains in HQ. The integration of genomic and transcriptomic tools for plague epizootic investigation provided more details about the evolution and phenotype mechanisms of *Y. pestis* than the traditional plague surveillance techniques. The use of these tools could become a new model for field investigations of plague, as well as for the surveillance of other animal-derived diseases.

## Data Availability Statement

The raw sequencing data and processed data of RNA-seq have been deposited in the NCBI Gene Expression Omnibus (GEO) under the accession number GSE161888. The whole genome sequencing data have been deposited in the NCBI Sequence Read Archive under BioProject ID PRJNA661196. The authors declare that all the data supporting the findings of this study are available within the article and supplementary information files or from the corresponding author upon reasonable request.

## Ethics Statement

Ethical review and approval was not required for the animal study because this research was done as part of routine plague surveillance.

## Author Contributions

LS, PW, and YC designed the research. LS, YG, PW, YZ, FY, HaZ, and SD performed the laboratory work and collected the data. JQ, HoZ, and CY participated in the analysis of the sequencing data. YW, GZ, YS, and RY contributed valuable technical expertise. LS, JQ, PW, and YC wrote the first draft of the manuscript and prepared figures. All authors took part in editing the manuscript, read and approved the final version.

## Conflict of Interest

The authors declare that the research was conducted in the absence of any commercial or financial relationships that could be construed as a potential conflict of interest.

## Footnotes

1 https://github.com/rrwick/Unicycler

2 http://tree.bio.ed.ac.uk/software/figtree/

3 http://www.applied-maths.com/bionumerics

4 http://www.ncbi.nlm.nih.gov/

5 http://asap.ahabs.wisc.edu/mauve/

6 https://www.bioinformatics.babraham.ac.uk/projects/fastqc/

7 https://www.uniprot.org/
