# Supplementary figures and images for: New Genotype of Yersinia pestis Found in Live Rodents in Yunnan Province, China
Source: Front Microbiol. 2021 Apr 15;12:628335. doi: 10.3389/fmicb.2021.628335 (PMC8084289; doi:10.3389/fmicb.2021.628335)

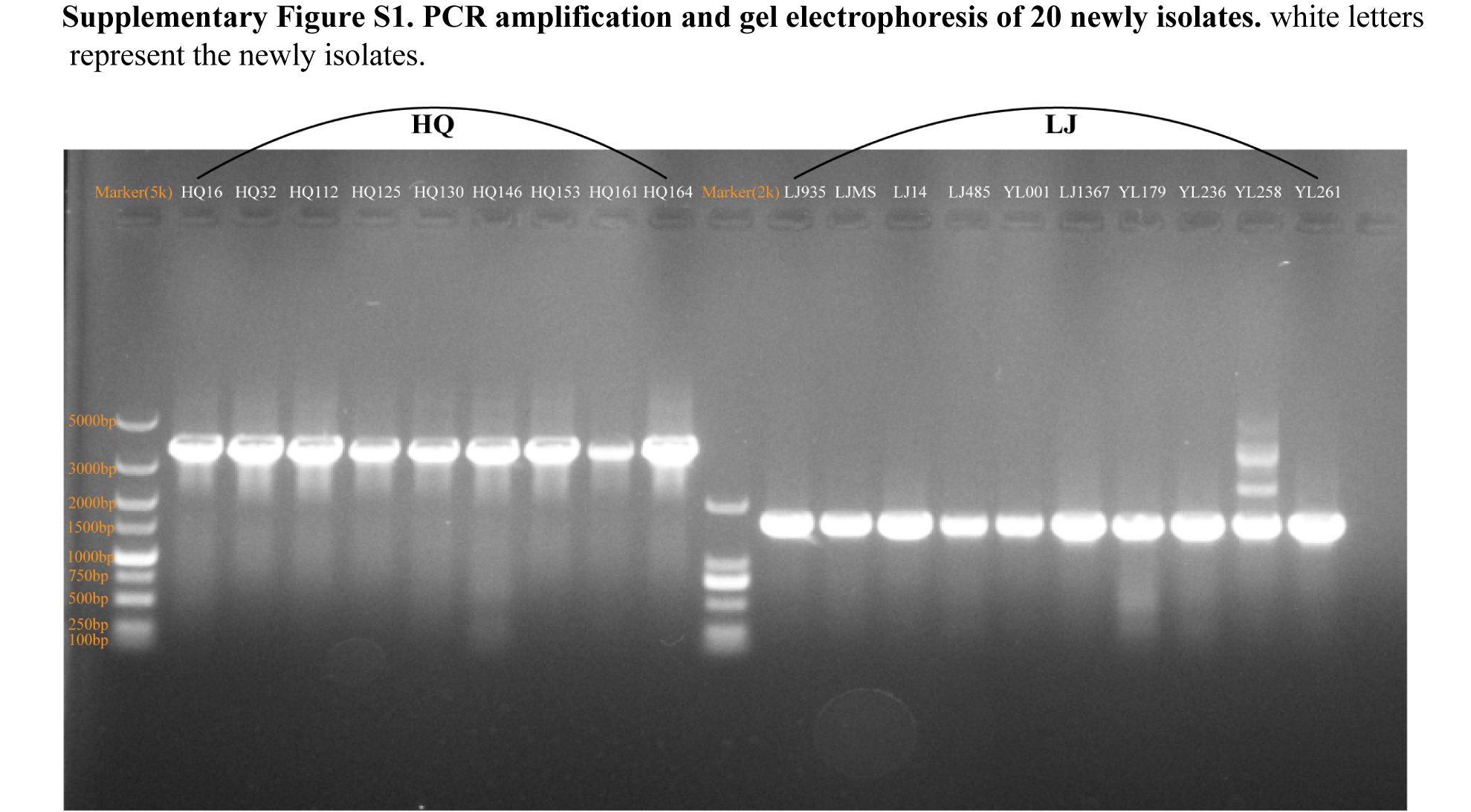

Supplement: Supplementary file 1 [file Image_1.TIF]

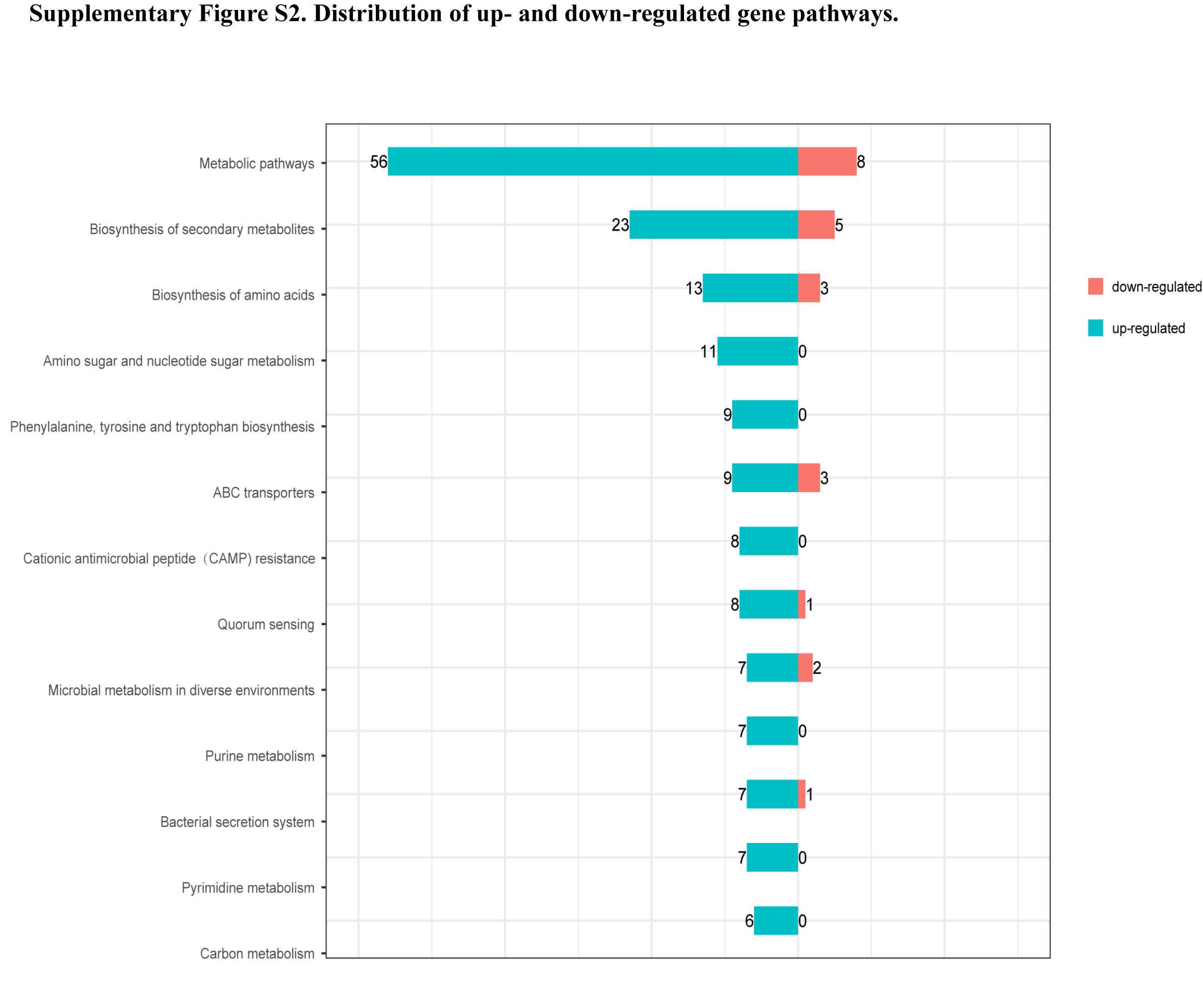

Supplement: Supplementary file 2 [file Image_2.TIF]
